# Synthesis of Porous Carbon Monoliths Using Hard Templates

**DOI:** 10.3390/ma9030214

**Published:** 2016-03-21

**Authors:** Olaf Klepel, Nina Danneberg, Matti Dräger, Marcel Erlitz, Michael Taubert

**Affiliations:** Brandenburg University of Technology Cottbus—Senftenberg, Faculty of Natural Sciences, POB 101548, Senftenberg D-01958, Germany; nina.danneberg@b-tu.de (N.D.); matti.draeger@b-tu.de (M.D.); marcel.erlitz@b-tu.de (M.E.); michael.taubert@b-tu.de (M.T.)

**Keywords:** carbon monolith, porous concrete, zeolite monolith, hard template

## Abstract

The preparation of porous carbon monoliths with a defined shape via template-assisted routes is reported. Monoliths made from porous concrete and zeolite were each used as the template. The porous concrete-derived carbon monoliths exhibited high gravimetric specific surface areas up to 2000 m^2^·g^−1^. The pore system comprised macro-, meso-, and micropores. These pores were hierarchically arranged. The pore system was created by the complex interplay of the actions of both the template and the activating agent as well. On the other hand, zeolite-made template shapes allowed for the preparation of microporous carbon monoliths with a high volumetric specific surface area. This feature could be beneficial if carbon monoliths must be integrated into technical systems under space-limited conditions.

## 1. Introduction

Porous carbon materials are promising candidates for manifold applications in gas separation technologies in the fields of energy conversion and storage, electrochemistry, and catalysis [1,2,3,4]. The long-standing interest in porous carbons can be explained by a number of outstanding features:

- the chemical stability against acids and bases;
- the temperature stability (in the absence of oxygen);
- the capability of being prepared with tailored pore systems over the whole range, from micro- up to macropores, while the pores can be arranged hierarchically; and
- the capability of being prepared as monoliths with defined geometric shape.

Especially due to the latter feature, preparation methods using hard templates have been proven as successful. The basic principle consists in the filling of pores of an inorganic material (*i.e.*, the template) with a carbon precursor, the carbonization of the precursor, and the removal of the template, e.g., by dissolving in hydrofluoric acid. As a result, the negative replica of the template is obtained. Templates commonly used are zeolites, silicas, porous glass, and porous concrete [5,6,7,8,9,10,11,12,13,14,15,16,17,18,19,20]. Among the discussed materials, porous concrete has been proven to be a most promising template. Porous concrete is advantageous over other templates because it is a low-cost construction material and is produced at an industrial scale. Furthermore, it is produced in monolithic form and can easily be shaped in any geometrical form using simple mechanic tools such as drills and saws [9,10]. So far, the production of cylinder and cubes has been reported [10]. The carbon replicas of porous concrete are mainly macroporous. They can easily be flown through by liquids, as is shown by the averaged Darcy permeability of 10 × 10^−12^ m^2^ [21]. Furthermore, they are able to bear a minimum mechanical pressure of 2.5 bar [21].

To increase the internal surface area and to obtain a hierarchical pore system, we first extended the template-assisted synthesis procedure by the simultaneous activation of the carbon matrix with zinc chloride [18]. In such a way, the Brunauer Emett Teller (BET) surface area could be increased from about 600 m^2^·g^−1^ up to about 1400 m^2^·g^−1^. We assumed that the pores formed by the activation are mainly located in the walls of the (primary) meso- and macropores that stems from the template. However, the activation occurs simultaneously to the carbonization, so it is impossible to separate the contribution of the individual factors, namely, the template effect and the activation processitself, to the development of the overall pore system. It was assumed that a part of the narrow pores created by the activating agent does not branch off from the (primary) meso- and macropores; on the contrary, narrow pores can block them as a bottleneck. That is why we decided to perform a post-synthesis activation using KOH as the activating agent. Here, the formation of the primary pore system was completed before the activation starts, and the understanding of the pore formation process was facilitated. Any pores created by the activation were expected to branch off from the primary pore system because the activating agent is most probably transported through these pores. It was therefore assumed that the resulting pore system would be hierarchically arranged. The carbon monoliths obtained that way are interesting, especially for dynamic adsorption systems; the micropores provide high surface areas the macropores are beneficial for the mass transport through the monoliths. Here, especially the removal of desorbed molecules must be mentioned because this step of technical processes can determine the practical applicability of the material to a great extent [20].

However, if porous monoliths are used as a construction component in technical devices, additional problems may arise. Due to spatial limitations in such technical systems, the geometric volume of the monoliths must not be too large, *i.e.* the volumetric specific surface area (given in m^2^ per m^3^ monolith volume) will likely be large. This requirement is hard to meet if the monolith—as is the case for porous concrete-derived carbons—contains macropores to a great extent. Therefore, besides porous concrete, other templates must be investigated. However, in comparison with porous concrete, the preparation of monoliths of other porous inorganic materials such as silica and zeolites is more difficult. Recently, the preparation of compact binderless zeolite shapes such as tubes and honeycombs has been reported [22]. Zeolites are interesting template materials to prepare microporous carbons [5]. Compared to binder-made shapes, these monoliths contain zeolite to a high degree and are likely promising materials for the template-assisted synthesis of porous carbon monoliths with enhanced raw density, *i.e.*, decreased geometric volume and high volumetric specific surface area.

Here, we report on recent attempts to prepare porous carbon monoliths with different defined geometric shapes such as cylinders, tubes, and thin plates. In the first part, novel materials obtained from porous concrete as the template with a hierarchical pore system will be presented. The origin of the pore system will be discussed in detail. In the second part, we describe the first attempts to produce porous carbon tubes using binderless-made zeolite tubes as a novel, hard template.

## 2. Materials and Methods

### 2.1. Materials

A technical product of porous concrete (Ytong DIN 4166, Xella Group, Duisburg, Germany) delivered as panels was used as the template raw material. From the panels, differently shaped monoliths were obtained by drilling and sawing. According to previously reported works [18], the template samples were treated with hydrochloric acid that dissolves the calcium and aluminum containing concrete phases. The template monoliths were impregnated in a vacuum desiccator with an aqueous solution of sucrose (68 wt %). The impregnated template samples were then calcined in a nitrogen stream at 873 K or 1173 K. After dissolving the template in hydrofluoric acid and rinsing in water, the replicas were obtained. A part of these char samples were then completely infiltrated with aqueous solutions of potassium hydroxide of concentrations in the range of 3 up to 20 molar. The according mass ratios of KOH to carbon were in the range from 0.8 to 4.8. The infiltrated samples were dried in an oven at 383 K overnight and then calcined in a two-step procedure at 573 K and 1073 K, respectively, in a nitrogen stream for three hours in each case. The heating rate was 10 K·min^−1^. Afterwards, the samples were again rinsed with water to remove all residues and reaction products of the activating agent.

The notation of the samples is demonstrated on the following examples: 873-3M refers to a sample that was carbonized at 873 K and treated with a 3-molar solution of potassium hydroxide. 873-0M refers to a blank sample that was carbonized at 873 K. If the whole series is mentioned, they are labeled as 873-*x* and 1173-*x*, respectively.

The synthesis of microporous carbon tubes was performed using novel Nay zeolite-made tubes (20 mm in length, and 6 and 3 mm in outer and inner diameter, respectively) whose synthesis procedure has been described very recently [22]. Further details are given in the Results and Discussion section. Different synthesis routes were applied to obtain carbon replicas. First, a route using sucrose as the carbon precursor was applied in accordance with the method described above. Furthermore, a preparation via chemical vapor deposition (CVD) using n-hexane as the carbon precursor was investigated. For this, the zeolite tube was placed in a flow reactor. The reactor was flown through by a gas stream consisting of nitrogen/n-hexane (15 mol % n-hexane) at 973 K. After cooling down in nitrogen, the zeolitic template was dissolved in hydrofluoric acid and rinsed with water.

### 2.2. Characterization Methods

Nitrogen adsorption and desorption isotherms were measured at 77 K on a Micromeritics ASAP 2020 volumetric adsorption system. The total surface area and micropore volume were determined using the BET equation and the method of Dubinin-Radushkevich, respectively. The total volume *V*_por total N2_ was calculated by the single point method at a relative pressure of 0.97 from the nitrogen adsorption isotherms. The pore size distribution was obtained from the adsorption branch of the nitrogen isotherm using the density functional theory (DFT, slit pores).

Carbon dioxide adsorption isotherms were measured at 273 K on a Micromeritics ASAP 2020 volumetric adsorption system in the pressure range up to 0.1 MPa, (corresponding to a relative pressure of *p*/*p*_0_ = 0.03).

The total volume *V*_por total H2O_ was obtained by a complete infiltration of the carbons with water in a vacuum (about 15 min) and subsequent weighing.

The macropore system of the carbons was investigated via mercury intrusion in the device Poremaster from Quantachrome. These measurements were done at the Institute for Technical Chemistry of the Leipzig University by the working group of D. Enke.

SEM photographs were taken on a FEI Nova Nanolab 200 (Portland, OR, USA) (accelerating voltage 10 kV) using a secondary electron detector. These measurements were done by the working group of Dirk Enke at Leipzig University.

## 3. Results and Discussions

### 3.1. Porous Concrete-Derived Monoliths

**Figure 1:**
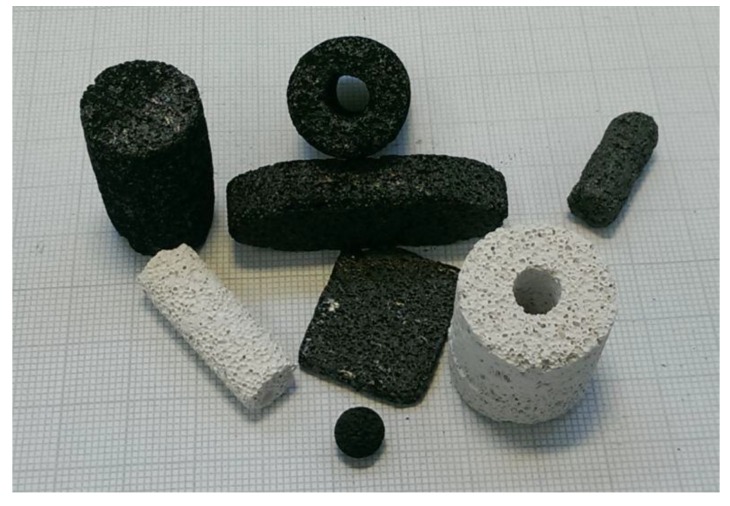
Carbon replicas and selected porous concrete-made template monoliths (one square of the pad corresponds to 10 mm).

After dissolution of the template, porous carbon monoliths in different shapes were obtained (Figure 1). Similar to the production of cubes and cylinders that has been reported so far [10], we first extended the variety of geometric shapes by thin plates, tubes, and spheres. The white colored monoliths represent a selection of the used template bodies.

**Figure 2:**
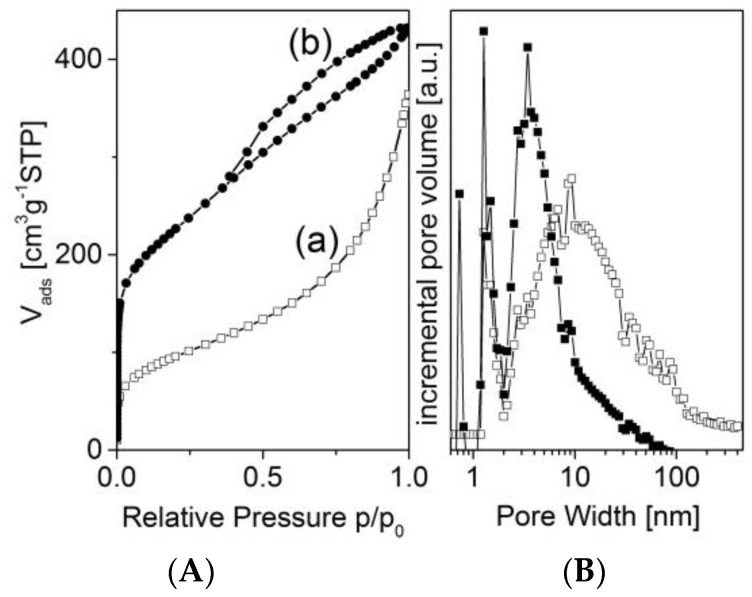
Nitrogen adsorption isotherms (**A**) and pore size distribution functions (DFT) (**B**) of the porous concrete-made template (a) and the carbon replica 873-0M (b).

**Figure 3:**
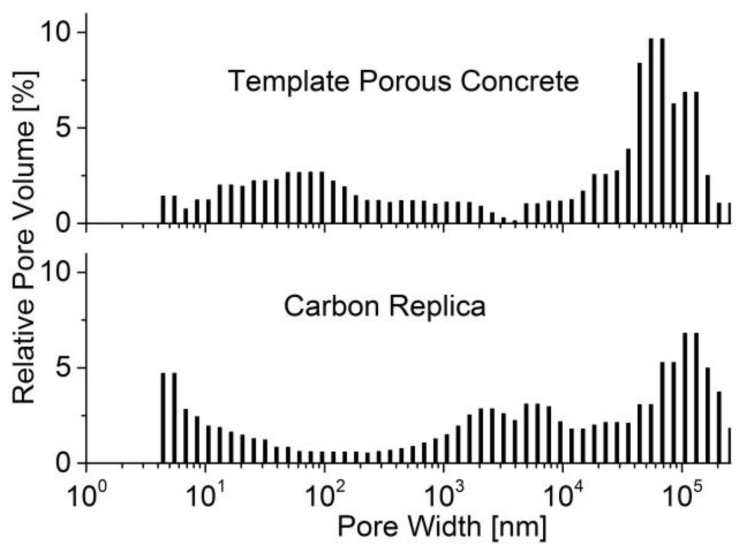
Pore size distribution (obtained by mercury intrusion) of the porous concrete-made template and the according carbon replica (carbonized at 873 K).

To understand the replication process in more detail, the pore systems of both the template and the carbon replica were investigated via nitrogen adsorption and mercury intrusion, respectively. (Figure 2 and Figure 3). The nitrogen adsorption isotherms and the according pore size distribution functions show that the template pores range at least from micro- up to mesopores. Furthermore, the uptake close to a relative pressure of 1 points to the existence of macropores. On the other hand, the replica pores in the micro- and mesopore range could be found (Figure 2). The origin of these pores will be discussed later in the context of the activating process.

Pore size distributions were investigated via mercury porosimetry (Figure 3). The template macropores in the range of about 30 up to 100 µm are obviously transferred to the carbon replica. The widths of these replica pores are defined by the thickness of the template pore walls and the width of unfilled template pores, respectively. This can be considered a hint that template macropores are not filled with carbon.

**Figure 4:**
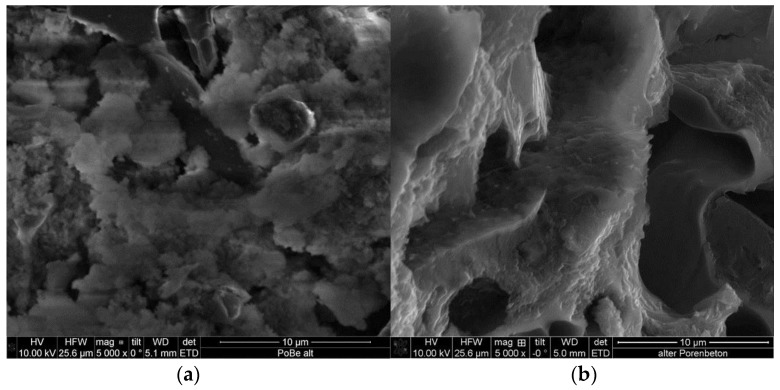
SEM photograph of porous concrete (**a**) and an according carbon replica (carbonized at 873 K) (**b**).

The smaller macropores of about 0.1 µm could be located in the foam-like areas, which are visible in the SEM photograph in Figure 4a. However, in the pore size distribution of the carbon replica, these pores are less pronounced. This is in accordance with the SEM photograph of the carbon where the foam-like domains are not as visible. Obviously, these parts of the template were not incorporated into the carbon replication process. After the template dissolution with HF, these areas disappeared and left porosity in the negative carbon replica. Therefore, new pores of about 5 µm in diameter were found in the carbon via both methods (Figure 3 and Figure 4).

The textural data of the used template are given in Table 1. The total pore volume was measured by different methods. As the nitrogen adsorption experiment produced a value of 0.56 cm^3^·g^−1^, other methods such as helium pyknometry and water infiltration produced values of about 2.5 cm^3^·g^−1^, which are considerably higher. As mainly micro- and mesopores are detected by the nitrogen adsorption method, one can conclude that the difference value of about 2 cm^3^·g^−1^ can be attributed to the macropore volume. However, the total pore volume obtained from the mercury porosimetry is somewhat lower than the values obtained by the water infiltration method and helium pyknometry. Obviously, a part of the porous concrete pores was connected by narrow pores that could be penetrated not by mercury but by water and helium. Therefore, care had to be taken when interpreting the pore size distribution of the porous concrete template in extensive detail.

There are distinct hints that the carbon is mainly formed in the micro- and mesopores. Only about 30% of the pore volume measured by nitrogen adsorption is infiltrated with carbon (Table 1). Furthermore, experiments applying repeated loading of template-carbon-composites with a precursor (sucrose) solution and subsequent carbonization show that only about two thirds of the template pore volume (estimated by nitrogen adsorption) can be filled with carbon. Considering this, we conclude that significant carbon deposition in the macropores should be excluded. Although macropores are initially filled with precursor, most of the precursor would be transported by capillary suction to the micro- and mesopores during the heat treatment.

This would be in accordance with the conclusion made above that mesopores (*i.e.*, pores considerably narrower than 0.1 µm) should be infiltrated by carbon.

To introduce a hierarchical pore system consisting in micro-, meso- and macropores, we then activated carbon cylinders via a treatment with KOH. The carbon monoliths preserved their shape during the activation procedure and were mechanical stable. The only exceptions were the samples from the series 1173-*x* (*i.e.*, carbonized at 1173 K), activated with the highest concentration of the KOH solution, *i.e.*, 20 molar. These monoliths collapsed partially.

**Figure 5:**
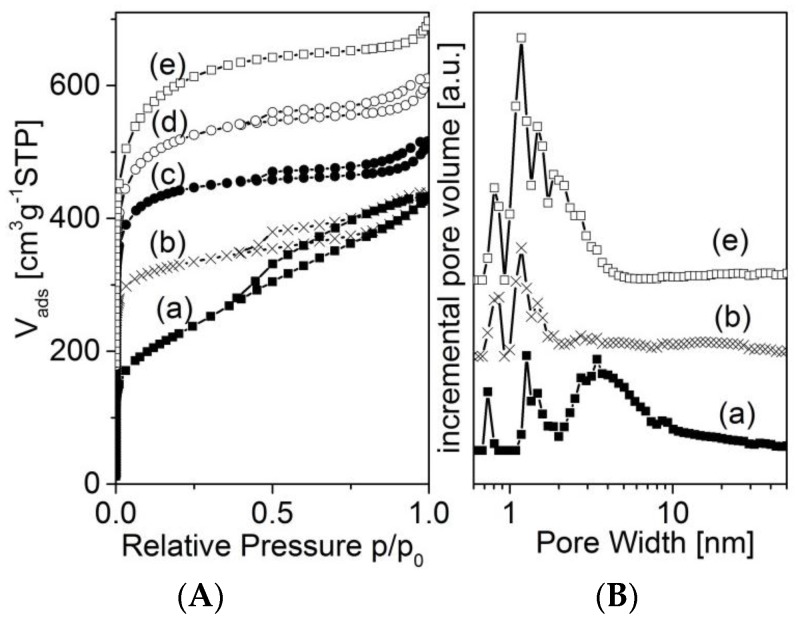
Nitrogen adsorption isotherms (**A**) of the samples 873-0M (a); 873-5M (b); 873-12M (c); 873-16M (d); 873-20M (e); and selected pore size distribution functions (**B**); the low pressure region is shown in the supplementary material (Figure S1).

**Figure 6:**
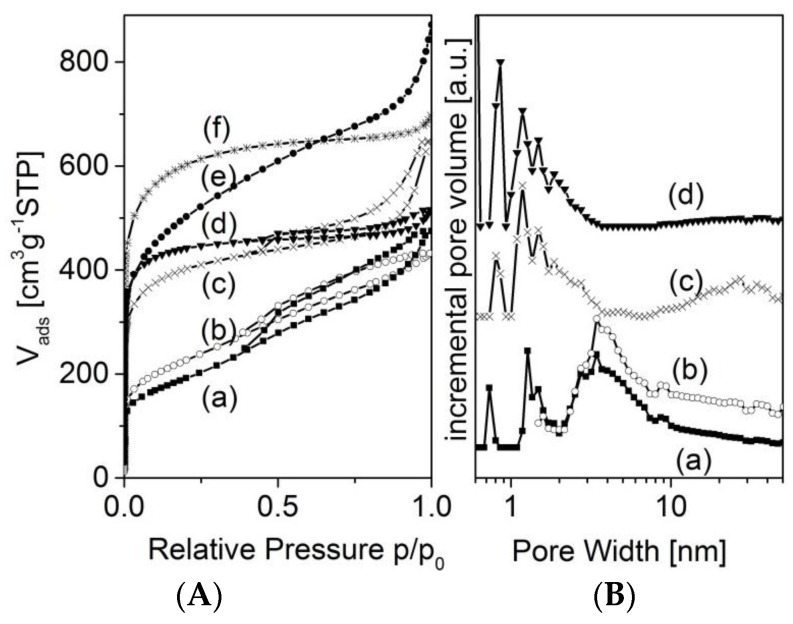
(**A**) Nitrogen adsorption isotherms of the samples 1173-0M (a); 873-0M (b); 1173-12M (c); 873-12M (d); 1173-20M (e); 873-20M (f); and selected pore size distribution functions (**B**).

Nitrogen adsorption isotherms of selected samples are shown in Figure 5 and Figure 6. All isotherms show a strong increase in adsorption at low relative pressure, indicating a high degree of microporosity. However, there are differences in the adjacent region of medium and higher relative pressure. As the blank samples 873-0M and 1173-1-0M show a rather continuous increase in adsorption, the activated carbons of the series 873-*x* reach almost a plateau. Here, the carbons treated with a KOH solution with a lower concentration show a sharp knee at a relative pressure of about 0.03 (Figure 5, curve (b)). The knee becomes more flat at a higher concentration of the activating agent (Figure 5, curves (d) and (e)). The associated pore size distribution functions (Figure 5B) illustrate that these materials are microporous to different degrees. Additionally, the non-activated sample 873-0M possesses mesopores in the range between 2 and about 10 nm. These mesopores disappear after the activation with a low-concentration KOH solution (5 M). Instead of the mesopores, more micropores between about 0.7 and 2 nm become visible. Applying the activation with a highly concentrated solution (20 molar) produces different effects. First, a further increase of microporosity is observed. Second, new pores in the region of larger micropores and smaller mesopores between about 1.5 and 3 nm are created.

The activated carbons of the series 1173-*x* show an additional increase of adsorption at higher values of relative pressure, which indicates the development of mesoporosity (Figure 6). These mesopores are in the range of about 30 nm (Figure 6B).

The micropore volume and the BET surface area as the main textural features correlate strongly with the concentration of the solution of the activating agent potassium hydroxide (Table 2 and Table 3). As the micropore volume reaches values up to about 0.8 cm^3^·g^−1^, the BET surface area could be increased to up to 2000 m^2^·g^−1^. Such values are the highest possible values for stable monoliths. They are in the range of surface areas of porous carbon monoliths, e.g., 1854, 1270, and 1090 m^2^·g^−1^, which have been reported in the literature [12,16,17]. A theoretical value of the carbon BET surface area was calculated from the textural properties of the template basing on the assumption of a common interface carbon/template (see Supplementary Materials). The value of 308 m^2^·g^−1^ is considerably lower than the values found for all investigated carbons. Even the BET surface area of the non activated carbons 873-0M and 1173-0M are more than twice as large.

The samples from the 873-K pretreated char possess higher values of both investigated parameters than the carbons of the series 1173-*x*. On the other hand, the total pore volume V_p/total/N2_ estimated by nitrogen adsorption is lower for the carbons of the 873-*x* series. Furthermore, it has been found that the mesopore volume V_p/meso/N2_ of the series 873-*x* decreases (in most of the cases) after the activation step. On the other hand, for the carbons of the series 1173-*x*, the mesopore volume remains quite constant; it only increases for the highly activated sample 1173-20M.

A theoretical value of the carbon total pore volume has been calculated from the textural properties of the template based on the assumption of a common interface carbon/template (see Supplementary Materials). It is very interesting that the calculated value of 7.3 cm^3^·g^−1^ is only met by the pore volume values V_p/total/H2O_ of the highly activated carbons (Table 2 and Table 3). For the starting materials 873-0M and 1173-0M, these values are considerably lower. The values of V_p/total/H2O_ become closer to the theoretical value with an increasing concentration of the activating agent.

**Figure 7:**
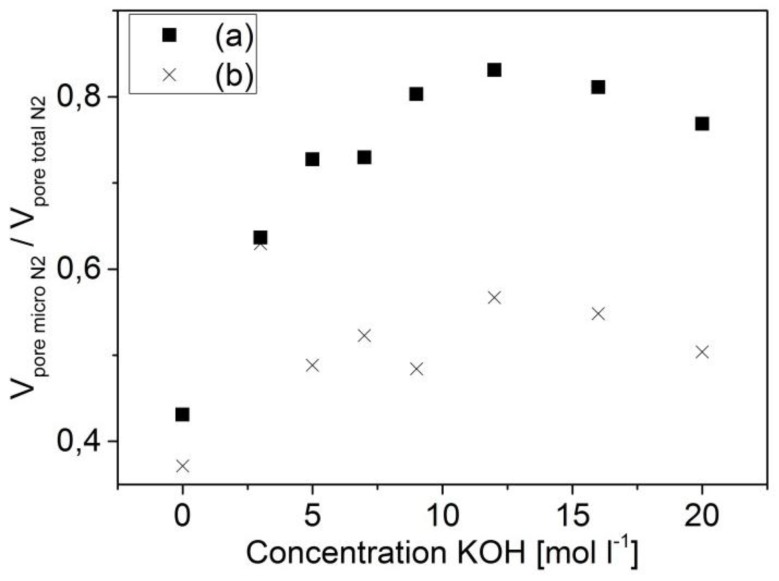
Ratio of micropore volume to total pore volume (micropore fraction) in dependence on the concentration of the used activation agent: series 873-*x* (a) and series 1173-*x* (b).

The overall difference in porosity between the two investigated series is reflected in the ratio of micropore volume to total volume V_p/micro_/V_p/total_ estimated from the nitrogen adsorption (Figure 7, Table 2 and Table 3). These values are considerably higher for the carbons of the 873-*x* series.

**Figure 8:**
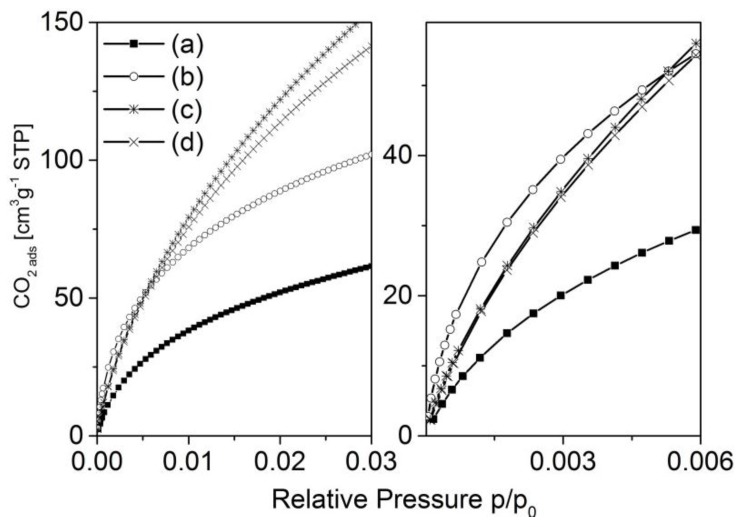
Carbon dioxide adsorption isotherms of the samples 873-0M (a); 873-3M (b); 873-12M (c); 873-16M (d).

**Figure 9:**
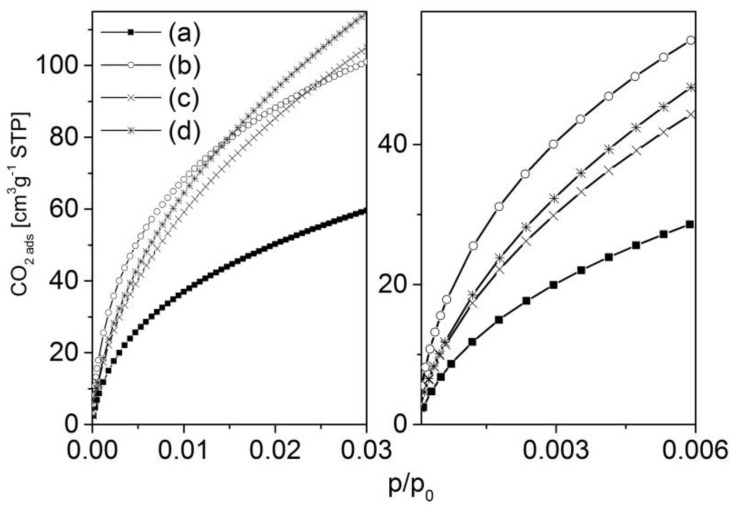
Carbon dioxide adsorption isotherms of the samples 1173-1-0M (a); 1173-1-3M (b); 1173-1-12M (c); 1173-1-16M (d).

Carbon dioxide adsorption experiments have been carried out in order to investigate the narrow micropores of the carbons. For both investigated series, the activated carbons show an increase in adsorbed carbon dioxide compared with the corresponding blank samples. It is interesting that, in the region of very low relative pressure, the strongest increase in adsorption is observed for the samples activated with the lowest concentration of potassium hydroxide (Figure 8 and Figure 9, right hand side). Here, with increasing concentration of potassium hydroxide, the amount of adsorbed carbon dioxide decreases. However, that situation changes at somewhat higher relative pressures of about 0.005 (series 873-*x*) and about 0.01 (series 1173-*x*), respectively. The isotherms cross each other and the amount of adsorbed carbon dioxide then correlates with the concentration of the activating agent.

To summarize and discuss the findings presented here, one must state that the development of the carbon pore system is controlled by a complex interplay of different processes and effects:

(i) the template effect;
(ii) gasification reactions during the carbonization process which cause inherent porosity; and
(iii) the action of the activating agent KOH.

To elucidate the origin of the pore system, one must separate the contribution of these effects. In the blank samples 873-0M and 1173-0M, the template effect should dominate the formation of the mesopores and the macropores. This can occur either by the direct effect, *i.e.*, the removal of template pore walls or by the transfer of empty template pores to the replica (Figure 3 and Figure 4). This has been proven in numerous investigations described in the literature [5,6]. However, the measured BET surface areas (676–800 m^2^·g^−1^) are more than two times larger than the theoretical value of 308 m^2^·g^−1^. From this, one can conclude that micropores (which provide most of the surface area) are formed not only by the direct template effect but also by gasification reactions to a considerable extent [23].

The activation procedure can effect the formation of micropores in the walls of the mesopores. This could explain the disappearance of the mesopores at about 4 nm of the activated samples during the simultaneous formation of new micropores (Figure 5 and Figure 6). If the walls of the mesopores are perforated by micropores to a great extent, they would not appear as mesopores anymore. However, there are differences between the two investigated series. For the 873-*x* materials, a direct correlation between the volume of carbon, which got lost during the activation procedure, and the created micropore volume was found (Figure S2). Obviously, these micropores are formed by a selective gasification of carbon species such as tar due to the action of KOH [24,25,26]. This correlation could not be found in samples carbonized at higher temperatures because here the volume of lost carbon exceeded the volume of the created micropore volume considerably (Figure S2). These carbons of the 1173-*x* series are carbonized to a greater extent. Their carbon species are therefore more akin in their nature and reactivity. The XRD patterns confirm a somewhat more pronounced graphite-like character or larger sized carbon domain of the carbon 1173-0M, which was carbonized at a higher temperature (Figure S3). As a consequence, these carbon species will be converted by the activating agent in a less selective way. This results in a more pronounced degradation of the solid carbon framework and finally in an enhanced discharge of carbon. This conclusion is supported by the creation of new mesopores at about 30 nm (Figure 6). Furthermore, the near constancy of the overall mesopore volume (Table 3) can also be interpreted as a hint to an enhanced degradation of the solid carbon framework: As the micropore formation is accompanied by the disappearance of mesopores (between 2 and 10 nm), the degradation of the carbon framework produces new mesopores at about 30 nm at the same time (Figure 6). These effects partially compensate each other and could be responsible for the apparent constancy of the overall mesopore volume.

The carbon dioxide adsorption isotherms also point to differences in the pore formation process (Figure 8 and Figure 9). In general, the low-pressure region of the adsorption isotherms reflects the adsorption in narrow micropores. As the strongest increase in adsorption is observed for the samples activated with the lowest concentration of potassium hydroxide, it is easy to assume that first micropores blocked by more reactive carbon species (e.g., tar) will be opened. At higher concentrations, carbon from the more solid skeleton (*i.e.*, the pore walls) will be gasified, resulting in a widening of micropores that is shown by an increase of carbon dioxide adsorption at a somewhat higher relative pressure. This effect is considerably more pronounced for the materials carbonized at lower temperatures (873-*x*), probably due to the lower degree of carbonization.

Besides this, the activating agent seems to expose blocked meso- or macropores. This would explain the steady increase of the total pore volume V_p/total/H2O_ with increasing concentration of KOH from about 4 cm^3^·g^−1^ up to values above 7 cm^3^·g^−1^, which are very close to the theoretical value of 7.3 cm^3^·g^−1^.

In summary, it can be stated that porous concrete is an excellent material for shaping templates for the production of porous carbon monoliths with defined geometry. As the template causes the formation of mainly macropores, an additional activation step can enhance the degree of micropores considerably, whereas the pores are most probably arranged hierarchically. In such a way, the advantage of enhanced mass transfer (provided by the macropores) could be combined with a high surface area due to a high degree of micropores.

### 3.2. Zeolite Based Carbon Monolith

As mentioned in the introduction section, the use of monoliths with high gravimetric specific surface area could be limited by the extent of their geometric volume. If the geometric volume is large, it could be difficult to integrate such monoliths into technical systems. In such a case, monoliths with high values of volumetric (instead of gravimetric) surface area are desired. This is impossible to achieve with porous concrete-derived templates because those replicas are mainly macroporous, *i.e.*, their geometric volume is too large.

We therefore tried to prepare carbon replicas of novel zeolite tubes whose preparation has been recently described [22]. These zeolite tubes were made from zeolite powder (NaY) that was mixed with a temporary binder (e.g., kaolinite). This mixture can be shaped by typical procedures such as extruding or casting. In a subsequent thermal and chemical treatment, the temporary binder will be converted into the according zeolite, *i.e.*, NaY. The resulting monolith consists of zeolite to a very high degree (XRD: 96% zeolite content). Investigations of different positions of the monolith could prove the homogeneous composition and crystallinity of the material. The monoliths are penetrated by macropores with a mean diameter of 0.3 µm, at which the fraction of mesopores is negligible [22].

**Figure 10:**
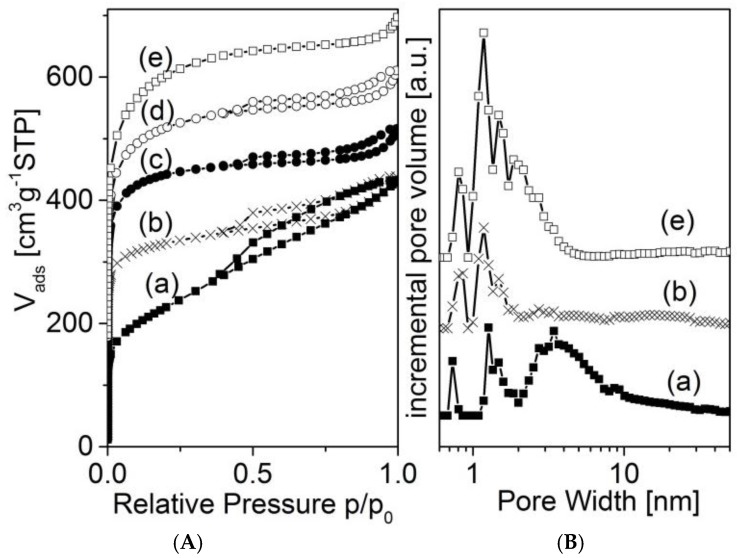
Nitrogen adsorption isotherms (**A**) and according pore size distribution functions (DFT) (**B**) of the zeolite-made tube (a) and the carbon replica tube (b) and, for comparison, of porous concrete-derived carbon 873-0M (c).

The textural data obtained by nitrogen adsorption are typical for NaY zeolite (Table 4, Figure 10). Mesopores could not be found.

**Figure 11:**
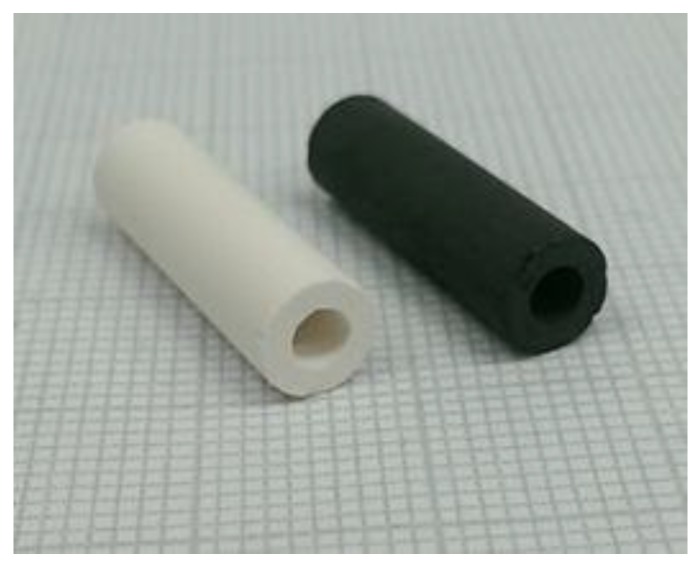
Binderless zeolite-made tube and the according carbon replica (one small square of the pad corresponds to 1 mm).

The preparation using sucrose as the precursor failed because the carbon monolith was not stable after the removal of the template. On the other hand, the synthesis applying the CVD method using n-hexane has been proven successful. After the dissolution of the template, porous carbon tubes that contain mainly micropores could be obtained (Figure 10 and Figure 11). The values of the volumetric BET surface area and the volumetric micropore volume are about two times larger than those of the porous concrete-derived materials (Table 5).

However, the theoretical value of the mass related BET surface area, which has been calculated from the textural properties of the template (see Supplementary Materials), is 1010 m^2^·g^−1^, *i.e.*, considerably higher than the measured value of 721 m^2^·g^−1^. A long-standing problem of such CVD methods is the interplay of mass transfer and chemical reactions (*i.e.*, carbon deposition) in the template pores, which may result in carbon gradients within the template monolith. As a consequence, the deposition should be applied at significantly low temperatures and for a long deposition time in order to favor the mass transfer against the reaction [27]. Our synthesis was performed at 973 K and with a 100-h deposition time. Despite this long duration, only 29% of the template pore volume was filled by carbon. Although this was sufficient to form a stable monolith, the value of the ratio of measured BET surface (721 m^2^·g^−1^) to the theoretical (predicted) surface area (1010 m^2^·g^−1^) is only 0.71. Such a ratio is considerably lower, as ratios that were found for replicas from zeolite powders at the same pore filling degree [23]. From that, one can conclude that parts of the template pores are blocked by carbon. The formation of a stable carbon network throughout the whole template monolith would therefore be hindered. The carbon network and consequently the pore structure of the replica would partially collapse, leaving wider pores. This conclusion is supported by the slope of the nitrogen adsorption isotherms at higher relative pressure, which indicates the existence of mesopores. However, mesopores are not to be expected for ideal replicas of zeolites. Thorough investigation of the macrokinetic of the deposition is still required to optimize the process and finally to improve the material and, especially, to shorten the deposition time. In particular, the choice of the carbon precursor is expected to play a crucial role.

## Figures and Tables

**Table 1:** Textural data of the porous concrete template and the carbon fraction of the template-carbon-composites.

BET Surface (m^2^·g^−1^)	*V*_p/micro_ (cm^3^·g^−1^)	*V*_p/total/N2_ (cm^3^·g^−1^) ^1^	*V*_p/total/H2O/He/Hg_ (cm^3^·g^−1^) ^2^	Carbon Fraction (cm^3^ Carbon/cm^3^ V_p/total/N2_) Series 873-*x*/1173-*x*
345	0.12	0.56	2.3/2.6/1.4	0.30/0.30

^1^ Single point at 0.99 p/p_0_, N_2_-adsorption; ^2^ H_2_O-infiltration/He-pyknometry/Hg-porosimetry.

**Table 2:** Textural data of carbons obtained from char carbonized at 873 K.

Textural parameter	873-0M	873-3M	873-5M	873-12M	873-16M	873-20M
BET surface (m^2^·g^−1^)	800	966	1117	1445	1761	2060
*V*_p/micro_ (cm^3^·g^−1^)	0.28	0.42	0.48	0.60	0.73	0.83
*V*_p/total/N2_ (cm^3^·g^−1^)	0.65	0.66	0.66	0.99	0.9	1.08
*V*_p/meso/N2_ (cm^3^·g^−1^) ^1^	0.37	0.24	0.18	0.39	0.17	0.25
*V*_p/micro_/V_p/totalN2_	0.43	0.64	0.73	0.61	0.81	0.77
*V*_p/total/H2O_ (cm^3^·g^−1^)	3.6	3.9	3.7	5.6	6.0	7.6

^1^ difference of V_p/totalN2_ and *V*_p/micro_ (cm^3^·g^−1^).

**Table 3:** Textural data of carbons obtained from char carbonized at 1173 K.

Textural parameter	1173-0M	1173-3M	1173-5M	1173-12M	1173-16M	1173-20M
BET surface (m^2^·g^−1^)	676	806	1038	1380	1260	1759
*V*_p/micro_ (cm^3^·g^−1^)	0.26	0.34	0.42	0.55	0.51	0.68
*V*_p/total/N2_ (cm^3^·g^−1^)	0.7	0.54	0.86	0.97	0.93	1.35
*V*_p/meso/N2_ (cm^3^·g^−1^) ^1^	0.44	0.20	0.44	0.42	0.42	0.67
*V*_p/micro_/V_p/totalN2_	0.37	0.63	0.49	0.57	0.55	0.50
*V*_p/total/H2O_ (cm^3^·g^−1^)	4.1	3.3	5.2	6.9	7.2	7.9

^1^ difference of *V*_p/totalN2_ and *V*_p/micro_ (cm^3^·g^−1^).

**Table 4:** Textural data of the zeolite tube.

BET Surface (m^2^·g^−1^)	*V*_p/micro_ (cm^3^·g^−1^)	*V*_p/total/N2_ (cm^3^·g^−1^)	Carbon Fraction (cm^3^ carbon/cm^3^ *V*_p/total/N2_)
667	0.31	0.32	0.29

**Table 5:** Specific mass related and volume related textural data of porous carbon monoliths.

Carbon Monolith	BET Area (m^2^·g^−1^)	BET Area (m^2^·cm^−3^)	*V*_p/micro_ (cm^3^·g^−1^)	*V*_p/micro_ (cm^3^·cm^−3^)	*V*_p/totalN2_ (cm^3^·g^−1^)	*V*_p/totalN2_ (cm^3^·cm^−3^)
Por. Concr. Replica	800	208	0.28	0.07	0.65	0.17
Zeolite Replica	721	430	0.28	0.17	0.44	0.26

## Supplementary Materials

The following are available online at www.mdpi.com/1996-1944/9/3/214/s1. Figure S1: Nitrogen adsorption isotherms (low-pressure region) of the samples 873-0M (a); 873-5M (b); 873-12M (c); 873-16M (d); 873-20M (e); and selected pore size distribution functions (right hand side). Figure S2: Comparison of the created micropore volume and the volume of gasified carbon for the chars pretreated at different temperatures; the straight line illustrates unity. Figure S3: X-ray diffraction patterns of the non activated carbon samples 1173-1-0M and 873-0M. Comment: Calculation of theoretical values of the BET surface area and the total pore volume.

## Abbreviations

The following abbreviations are used in this manuscript:

BET: Brunauer Emett Teller
CVD: Chemical vapor deposition
