# Supplementary material for: Synthesis of Porous Carbon Monoliths Using Hard Templates
Source: Materials (Basel). 2016 Mar 21;9(3):214. doi: 10.3390/ma9030214 (PMC5456719; doi:10.3390/ma9030214)
Supplement: Supplementary file 1 [file materials-09-00214-s001.pdf]

Supplementary Materials: Synthesis of Porous Carbon Monoliths Using Hard Templates

Olaf Klepel, Nina Danneberg, Matti Dräger, Marcel Erlitz and Michael Taubert

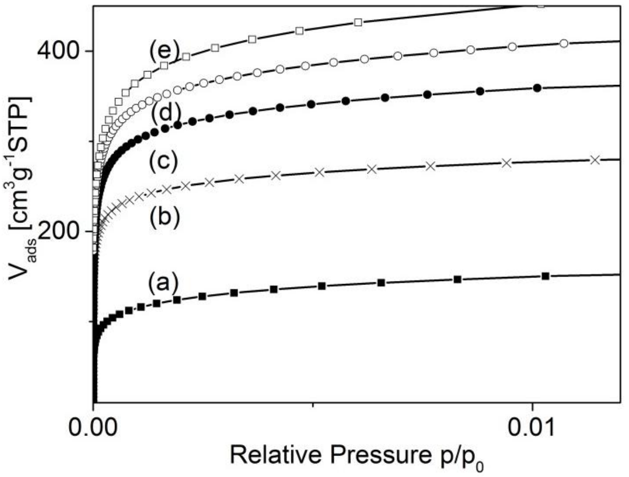

Figure S1. Nitrogen adsorption isotherms (low pressure region) of the samples 873-0M (a), 873-5M (b), 873-12M (c), 873-16M (d), 873-20M (e) and selected pore size distribution functions (right hand side)

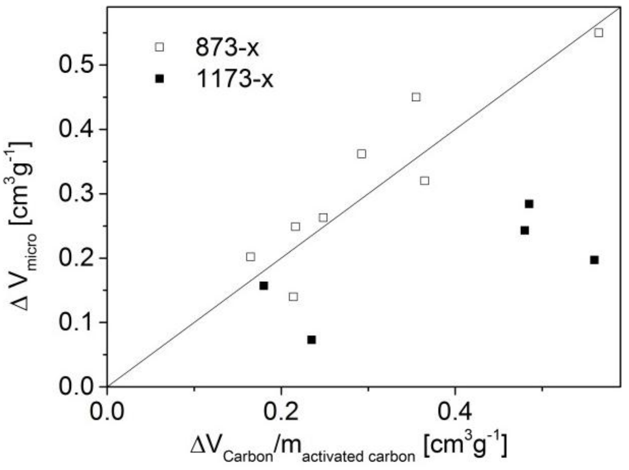

Figure S2. Comparison of the created micropore volume and the volume of lost carbon for the chars pretreated at different temperatures; the straight line illustrates unity.

The carbon loss due to the activation step was determined by weighing.

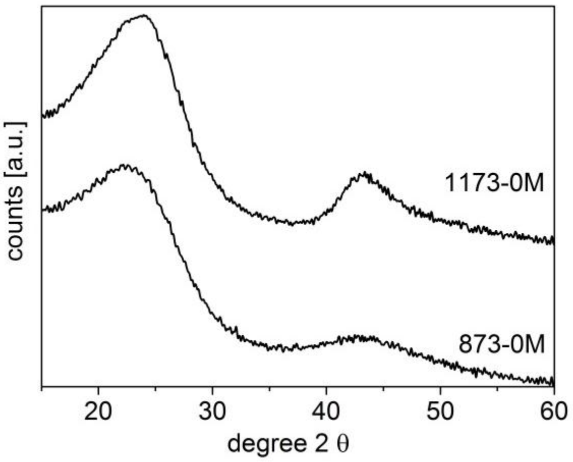

Figure S3. X-ray diffraction patterns of the non activated carbon samples 1173-1-0M and 873-0M.

The crystalline structure of the materials was investigated by X-ray diffraction (XRD) with a Bruker D2 Phaser instrument (Madison, WI, USA). Cu Kα radiation (wavelength λ = 1.5418 Å) was used as an X-ray source. The determination of graphitic and amorphous carbon structures was performed in the 2 θ angle range of 1–60° and with a step of 0.02° per second.

Comment: *Theoretical value of the carbon BET surface area and the pore volume (according [27])*

A direct template effect was defined as follows:

- the template pores and the carbon network have a common interface and so the internal surface area of the replica corresponds to those of the template;
- the pore sizes of the replica are determined by the thickness of the template pore walls;
- the pore volume stems by the skeleton volume of the template.

Assuming the direct template effect, the surface area of the carbon replica can be calculated from the template surface area and the mass of infiltrated carbon according

$$S_{theo} = \frac{S_{\text{template}}}{V_{\text{pore template}} \cdot \rho_{\text{skel carbon}}} \quad \text{(S1)}$$

The symbols are:

S_{theo}: calculated surface area of the carbon replica in m² per g carbon,

S_{template}: surface area of the template in m² per g template,

$V_{\text{pore template}}$: template pore volume in cm³ per g template,

$\rho_{\text{skel carbon}}$: skeleton density of nonporous carbon which is assumed to be 2.0 g carbon per cm³ carbon *i.e.*, somewhat lower than that of pure graphite.

From this, a ratio of the measured surface area and theoretical carbon surface area $S_{\text{measured}}/S_{\text{theo}}$ can be calculated. Any deviations from unity can be explained in terms of scenarios which differ from the direct template effect. If the ratio of $S_{\text{measured}}/S_{\text{theo}}$ is lower than unity, the carbon network, which has been formed in the template pores, was obviously not stable enough and collapsed (at least) partially. In case of ratios larger than unity, an inherent porosity of the carbon, mostly formed by gasification reactions, must be assumed.

In the same way, the theoretical value of the pore volume of the carbon replica can be calculated by

$$V_{\text{pore theo}} = \frac{V_{\text{skel template}} + V_{\text{pore template}} - x \cdot V_{\text{pore template}}}{m_{\text{carbon infil}}} \quad \text{(S2)}$$

Here the symbols are:

$V_{\text{pore theo}}$: theoretical value of the pore volume given in cm^3 per g carbon,

$V_{\text{skel template}}$: template skeleton volume in cm^3 per g template,

$V_{\text{pore template}}$: template pore volume in cm^3 per g template,

x: template pore filling degree expressed as the fraction of the carbon filled template pore volume in cm^3 carbon per cm^3 pore volume

$m_{\text{carbon infil}}$: mass fraction of infiltrated carbon in g carbon per g template.

From this, again, a ratio of the measured value and the theoretical value can be calculated. If the ratio of $V_{\text{pore measured}}/V_{\text{pore theo}}$ is lower than unity, different scenarios must be considered:

- the carbon particle got broken (due to low carbon content in the template),
- large unfilled template regions which would cause the formation of macropores in the replica; these pores cannot be detected by the applied adsorption method;
- blocked pores in the replica which cannot be infiltrated by any probe molecule.
